# NiNC Catalysts in CO_2_-to-CO Electrolysis

**DOI:** 10.1007/s40820-024-01595-y

**Published:** 2024-12-26

**Authors:** Hao Zhang, Menghui Qi, Yong Wang

**Affiliations:** 1 Department of Chemical Engineering, Massachusetts Institute of Technology, Cambridge, MA 02139 USA; 2 Department of Chemistry, University of Oxford, Oxford, OX1 3TA UK; 3 Advanced Materials and Catalysis Group, Department of Chemistry, Institute of Catalysis, Zhejiang University, Hangzhou, 310058 People’s Republic of China

**Keywords:** NiNC catalysts, CO_2_-to-CO electrolyzer, Faradaic efficiency, Carbon crossover coefficient (CCC), Mesoporous structures

## Abstract

CO_2_-to-CO electrolyzer technology converts carbon dioxide into carbon monoxide using electrochemical methods, offering significant environmental and energy benefits by aiding in greenhouse gas mitigation and promoting a carbon circular economy. Recent study by Strasser et al. in Nature Chemical Engineering presents a high-performance CO_2_-to-CO electrolyzer utilizing a NiNC catalyst with nearly 100% faradaic efficiency, employing innovative diagnostic tools like the carbon crossover coefficient (CCC) to address transport-related failures and optimize overall efficiency. Strasser’s research demonstrates the potential of NiNC catalysts, particularly NiNC-IMI, for efficient CO production in CO_2_-to-CO electrolyzers, highlighting their high selectivity and performance. However, challenges such as localized CO_2_ depletion and mass transport limitations underscore the need for further optimization and development of diagnostic tools like CCC. Strategies for optimizing catalyst structure and operational parameters offer avenues for enhancing the performance and reliability of electrochemical CO_2_ reduction catalysts.

## Highlights

- NiNC catalysts achieve nearly 100% faradaic efficiency in CO_2_-to-CO conversion.
- The carbon crossover coefficient is introduced as a diagnostic tool for performance optimization.
- Tandem electrolyzer design and mesoporous structures enhance product yields and efficiency.

## Graphical abstract

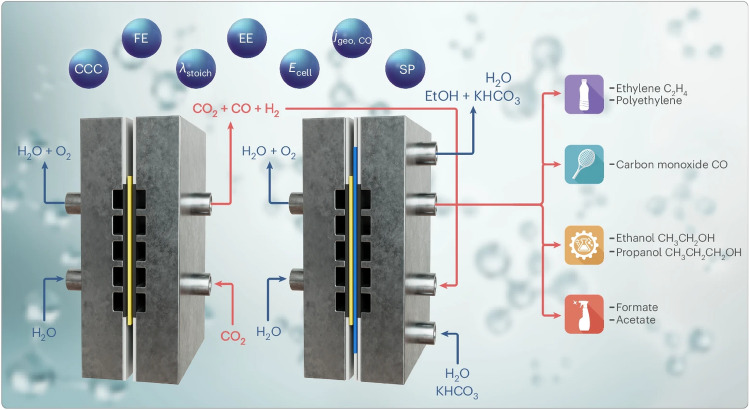

## Discussion

The CO_2_-to-CO electrolyzer is a device that utilizes electrochemical methods to reduce carbon dioxide (CO_2_) into carbon monoxide (CO). This technology holds significant environmental and energy application potential as it can convert CO_2_ into useful carbon resources, thereby aiding in mitigating greenhouse gas emissions and promoting a carbon circular economy [1]. In CO_2_-to-CO electrolyzers, catalysts are typically employed to facilitate the electrochemical reduction reaction of CO_2_, generating CO as the primary product [2]. These catalysts are usually metallic or carbon-based materials with efficient CO_2_ conversion performance. The design and operating conditions of the electrolyzer, such as current density, temperature, and electrolyte composition, are crucial for its performance and stability. With the increasing demand for CO_2_ reduction and renewable energy utilization, CO_2_-to-CO electrolyzer technology has attracted growing attention and research.

A recent paper by Strasser et al. published in *Nature Chemical Engineering* presents the design and diagnostic analysis of a high-performance CO_2_-to-CO electrolyzer cell [3]. The cell features a nickel–nitrogen-doped carbon (NiNC) catalyst in a pH-neutral, zero-gap configuration, demonstrating nearly 100% CO faradaic efficiency at current densities up to 250 mA cm^−2^, with 40% total energy efficiency and stable operation over 100 h.

**Fig. 1:**
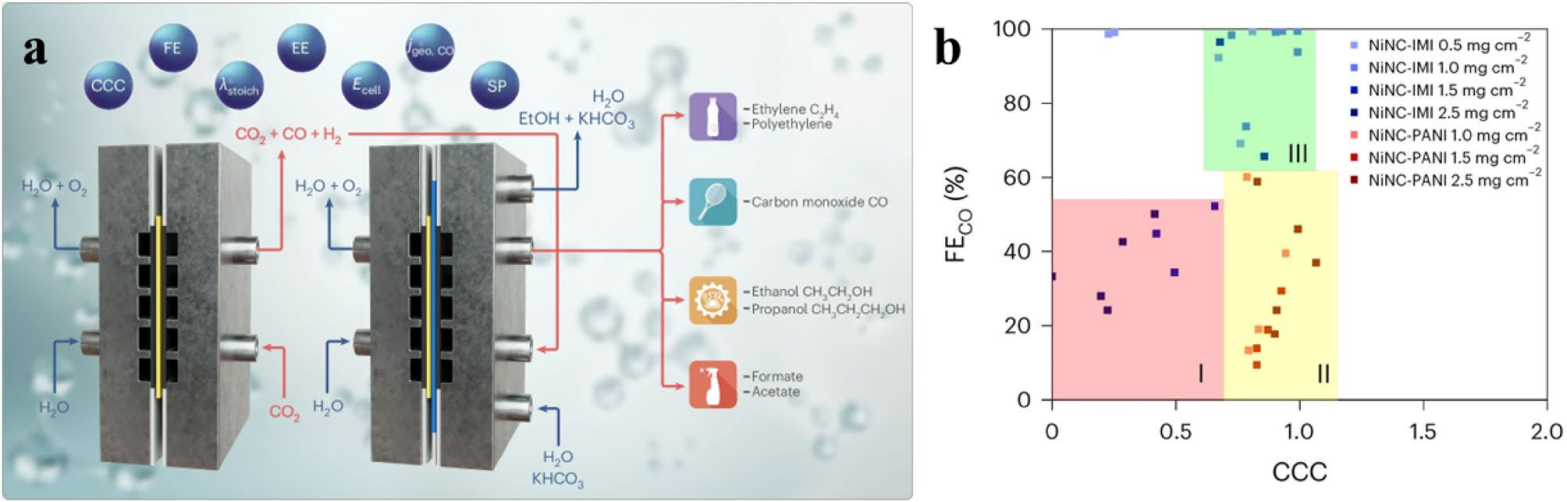
**a** CO_2_ valorization using coupled ‘tandem’ electrolyzer cells. **b** Cell diagnosis using the CCC and FE_CO_. FE_CO_ versus CCC plots yield distinct operating reaction transport regimes (red (I), yellow (II) and green (III)) to diagnose the mass transfer limitations. Reprinted with permission from Ref. [3]. Copyright 2024, Springer Nature Group. (Color figure online)

This study designs and diagnoses a high-performance CO_2_-to-CO electrolyzer, utilizing a nickel–nitrogen-doped carbon (NiNC) catalyst, achieving nearly 100% faradaic efficiency. Operating at a low stoichiometric CO_2_ excess ratio (λ_stoich_ ~ 1.2), the cell achieves a high molar CO concentration (~ 70 vol%) in the exit stream with a single-pass CO_2_ conversion rate of 40%. The study introduces the carbon crossover coefficient (CCC) as a diagnostic tool to quantify non-catalytic CO_2_ consumption and transport-related failures, providing insights into ionic transport mechanisms and undesired CO_2_ losses, diagnosing transport failures caused by salt precipitation and redissolution. Additionally, the proposed tandem cell design uses a CO_2_-to-CO electrolyzer to supply CO-rich streams to a second cell for the production of C_2+_ chemicals and fuels, aiming to improve overall efficiency and product yields by optimizing individual process steps (Fig. 1a).

The experimental methodology involves synthesis, characterization, and performance comparison. Initially, a series of NiNC catalysts were synthesized through the pyrolysis of various nickel salt precursors. Techniques such as powder X-ray diffraction, transmission electron microscopy, and X-ray photoelectron spectroscopy were employed to evaluate their chemical state, composition, morphology, porosity, and microstructure. Subsequently, the performance of the NiNC catalyst was evaluated and normalized by near-surface NiN_*x*_ site density (SD), which surprisingly illustrated almost identical intrinsic turnover frequency (TOF) for cell potential (E_cell_) > − 0.75 V. This suggested the NiN_*x*_ motifs is the active site for the CO_2_ to CO reaction and the catalytic performance is insensitivity of the emerging active site during pyrolysis to the chemical nature of the precursors. Significant TOF-E_cell_ difference was observed for E_cell_ < − 0.75 V, which is speculated that the sites accessibility is different. For a more in-depth analysis, the representative Ni-IMI with low loading outperform the NiNC-PANI especially in terms of CO mass activity, which can be explained by the mesoporous peculiarity of NiNC-IMI offers enhanced site accessibility, relieving the mass transport resistance. Despite the encouraging results, the study underscores the challenges in achieving commercial-grade performance, particularly in terms of mass transfer and accessibility of active sites.

The NiNC catalyst exhibited remarkable faradaic efficiency for CO production at neutral pH; while, the cell’s design facilitated operation with low stoichiometric CO_2_ excess, enhancing CO concentration in the exit stream and overall efficiency. Additionally, the development of reliable diagnostic tools for the analysis of the dynamic operation, transport processes and degradation at the electrode level of CO_2_-to-CO electrolyzers is becoming of equal priority. Strasser derived and defined the CCC value using a mathematical correlation between the catalytic production rates of CO (*V*_CO_) and H_2_ (*V*_H2_) as well as the CO_2_ input feed (*V*_CO2_, in) and the exit flow rate (*V*_out_). The proposed CCC value can be interpreted as the ratio between the rate of non-catalytic acid–base CO_2_ consumption and the rate of catalytic alkalinity production. This value is empirically measurable, unaffected by the Faradaic value, and elucidates the nature of the predominant anion traversing the membrane in a lucid manner: An experimental CCC value of 0 signifies the exclusive transfer of OH^−^ ions, a CCC value of 1 signifies the exclusive transport of CO_3_^2−^ ions, and a CCC value of 2 suggests the pure crossover of HCO_3_^−^ ions. Non-integer CCC values suggest the formation of non-stoichiometric (bi) carbonate species within the catalyst layer, coupled with the transport of mixed anions. This implies that only a fraction of the produced alkalinity (OH^−^) is converted into carbonate (with a CCC slightly below 1). If the carbonate is not swiftly transported away, it may subsequently react with another CO_2_ molecule to form bicarbonate (with a CCC slightly above 1). By utilizing the aforementioned analysis, researchers can obtain a dependable diagnostic tool for discerning the dynamic operations and transport processes by calculating the CCC value.

Strasser et al. identify three operational regions (green (III), yellow (II), red (I)) tied to in-plane (IP) and through-plane (TP) transport and CO_2_ conversion (Fig. 1b). For NiNC-IMI cells with high loadings (red region), low CCC and FECO reveal inadequate IP and TP transport. In contrast, NiNC-PANI cells with CCC ~ 1 and FECO < 60% show inefficient IP CO_2_ transfer to active sites. Optimal performance (green region) occurs at CCC ~ 1 and high FECO, as in NiNC-IMI cells at 1 mg cm^−2^, where effective site accessibility and transport are evident. CCC analysis reveals NiNC-IMI layers hinder CO_2_ access at higher loadings, unlike NiNC-PANI, which maintains accessibility.

Despite the high activity of NiNC-IMI, differences in site accessibility may lead to localized CO_2_ depletion, particularly under high loadings, resulting in a plateauing relationship between TOF and E_cell_. Additionally, increasing the thickness and density of the catalyst layer may hinder CO_2_ diffusion, especially in the smaller pores of NiNC-PANI, thereby affecting its performance [2]. As the catalyst loading increases, the activity of both NiNC-IMI and NiNC-PANI catalysts may decline due to mass transport limitations [4]. Moreover, localized CO_2_ depletion may occur during the catalytic process, leading to insufficient CO_2_ supply in certain areas and thereby reducing reaction efficiency. The significant differences in catalyst performance under different electrolyte environments present another crucial challenge, necessitating optimized catalyst designs to accommodate various operational conditions [5]. Although CCC has been proposed as a diagnostic tool, further research and validation are necessary to better understand and address the degradation mechanisms of catalysts. These drawbacks underscore the key challenges that need to be overcome in further developing and optimizing electrochemical CO_2_ reduction catalysts.

By optimizing the mesoporous structure to enhance site accessibility and reduce local CO_2_ depletion, refining catalyst layer thickness and porosity to ensure better CO_2_ diffusion and consistent performance at higher loadings, developing and validating diagnostic tools such as the CCC to better understand and improve transport properties and identify degradation regimes, and optimizing operational parameters such as the stoichiometric CO_2_ ratio to maximize CO production and overall cell efficiency, these measures highlight the strengths of the current catalysts and suggest avenues for enhancing their performance and reliability in electrochemical CO_2_ reduction applications.
